# SERIES: eHealth in primary care. Part 1: Concepts, conditions and challenges

**DOI:** 10.1080/13814788.2019.1658190

**Published:** 2019-10-10

**Authors:** Rianne M.J.J. van der Kleij, Marise J. Kasteleyn, Eline Meijer, Tobias N. Bonten, Elisa J.F. Houwink, Martine Teichert, Sanne van Luenen, Rajesh Vedanthan, Andrea Evers, Josip Car, Hilary Pinnock, Niels H. Chavannes

**Affiliations:** a Department of Public Health and Primary Care, Leiden University Medical Centre, Leiden, The Netherlands;; b Department of Clinical Pharmacy and Toxicology, Leiden University Medical Centre, Leiden, The Netherlands;; c Department of Clinical Psychology, Faculty of Social Sciences, Institute of Psychology, Leiden University, Leiden, The Netherlands;; d Department of Population Health, Section for Global Health, NYU School of Medicine, New York, NY, USA;; e Department of Health, Medical and Neuropsychology, Faculty of Social Sciences, Institute of Psychology, Leiden University, Leiden, The Netherlands;; f Centre for Population Health Sciences, Lee Kong Chian School of Medicine, Nanyang Technological University, Singapore, Singapore;; g Allergy and Respiratory Research Group, Usher Institute of Population Health Sciences and Informatics, The University of Edinburgh, Edinburgh, Scotland

**Keywords:** eHealth, primary care, vision, challenges

## Abstract

Primary care is challenged to provide high quality, accessible and affordable care for an increasingly ageing, complex, and multimorbid population. To counter these challenges, primary care professionals need to take up new and innovative practices, including eHealth. eHealth applications hold the promise to overcome some difficulties encountered in the care of people with complex medical and social needs in primary care. However, many unanswered questions regarding (cost) effectiveness, integration with healthcare, and acceptability to patients, caregivers, and professionals remain to be elucidated. What conditions need to be met? What challenges need to be overcome? What downsides must be dealt with? This first paper in a series on eHealth in primary care introduces basic concepts and examines opportunities for the uptake of eHealth in primary care. We illustrate that although the potential of eHealth in primary care is high, several conditions need to be met to ensure that safe and high-quality eHealth is developed for and implemented in primary care. eHealth research needs to be optimized; ensuring evidence-based eHealth is available. Blended care, i.e. combining face-to-face care with remote options, personalized to the individual patient should be considered. Stakeholders need to be involved in the development and implementation of eHealth via co-creation processes, and design should be mindful of vulnerable groups and eHealth illiteracy. Furthermore, a global perspective on eHealth should be adopted, and eHealth ethics, patients’ safety and privacy considered.

## Introduction

KEY MESSAGES

- eHealth should support the transition towards personalized medicine, self-management and shared decisions in primary care.
- Several conditions need to be met to ensure that eHealth applications are safe, evidence-based and of high quality.
- Innovative but valid research methodology—e.g. adaptive (action research) designs—is a prerequisite for ongoing success and sustainability of eHealth.

The number of consultations in primary care is steadily increasing [1,2], while clinical capacity is declining [3]. Moreover, the ageing patient population and the rise in patients presenting with multimorbidity put an ever-growing burden on primary care [4,5]. By 2030, there will be a global deficit of about 18 million—mostly primary care—health professionals (midwives, nurses, and physicians). In the UK, 8000 new full-time equivalent primary care clinicians are needed to respond to the pressing demands of primary healthcare, such as retirement and increased complexity of the workload [3]. Not only are the number of patients and their medical complexity changing, but also the patient-provider relationship is transforming. Furthermore, a shift towards personalized healthcare and supported self-management is occurring: patients are increasingly considered as equal partners in the therapeutic relationship and they want to be informed and take part in the medical decision-making process [6]. As a result, primary care providers are under growing pressure to provide high quality and accessible care that is, above all, cost-effective. National and international organizations, therefore, urge primary care providers to adopt new and innovative ways of working, including those incorporating eHealth [7–9].

**An everyday case**

Imagine a man in his mid-sixties, named Jon. Jon suffers from multimorbidity. Apart from end-stage chronic obstructive pulmonary disease (COPD), Jon has diabetes mellitus type 2 (DM2) and hypertension. He is also tobacco-dependent. Jon experiences a high burden of disease, and managing his disease remains challenging. Despite the medication instruction provided by his nurse practitioner, Jon is not able to use his medication correctly. Now, what if his nurse practitioner could combine her face-to-face instructions with a ‘smart’ inhaler or a ‘smart’ insulin pen that provides feedback on his medication use and technique?

Moreover, what if she could equip Jon with a tablet device and compatible wearables, giving him access to several lifestyle management apps (e.g. ‘*My Quit Coach*’)? These applications could also help him track his health parameters and symptoms and potentially improve his disease self-management skills. Social support is furthermore of vital importance to decrease disease burden and optimize disease management. What if the tablet device provided would allow him to stay in contact with his family via Skype, or establish a secure video connection with his nurse to discuss concerns?

The case of Jon illustrates how eHealth could help overcome some of the challenges primary care faces as it cares for people with complex medical and social care needs. However, there still are many unanswered questions. How (cost) effective is the application and integration of eHealth in primary care? How acceptable is it to patients, their caregivers, and primary care clinicians? What conditions need to be met and what challenges need to be overcome to ensure that eHealth lives up to its potential? And are there downsides to the application of eHealth in primary care?

Providing insight into the role eHealth could play in primary care, is the aim of a series on eHealth in primary care, which we will publish in the *European Journal of General Practice*. The series covers seven themes:

1. eHealth for primary care (this paper): concepts, conditions, and challenges
2. eHealth for primary care: addressing the ethical implications
3. Evidence-based eHealth for primary care: inclusive, individualized and blended
4. Implications of eHealth for primary care in medical education and vocational training
5. Implementation of eHealth and integration into primary care routines
6. Developing an eHealth infrastructure: the role of primary care providers
7. Global perspective: eHealth for primary care in low resource settings

## Concepts

### What is eHealth?

**Figure 1.**
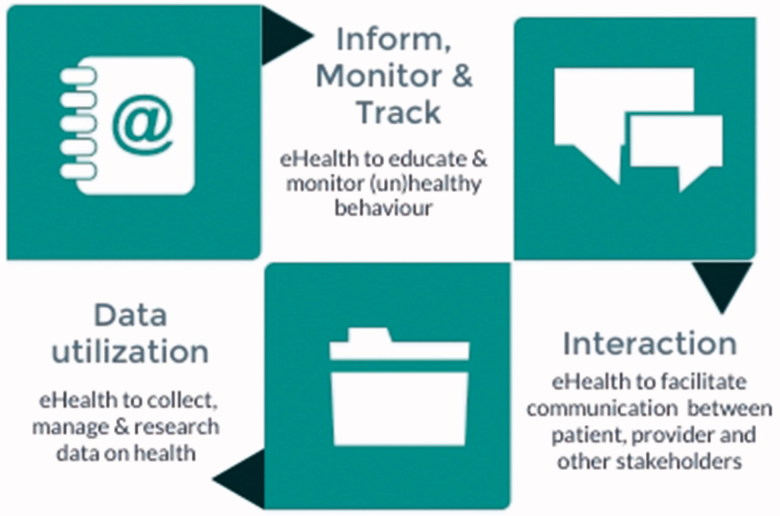
Conceptualization of eHealth in primary care, derived from Shaw et al. [13].

In the last decade, various definitions of eHealth have been proposed, and no consensus has yet been reached [10]. The most frequently cited definition is that of Eysenbach [11]: ‘eHealth is referring to health services and information delivered or enhanced through the Internet and related technologies. In a broader sense, the term characterizes not only a technical development, but also a state-of-mind, a way of thinking, an attitude, and a commitment for networked, global thinking, to improve healthcare by using information and communication technology.’ Although this definition is informative, a more elaborate description of eHealth is warranted. The conceptualization of eHealth, for instance, as provided by Shaw et al. [12], demarcates three distinct functions of eHealth, and details how they can contribute to primary care. The first function is ‘inform, monitor and track’, encompassing the use of eHealth technologies to observe and study health parameters. The second function of eHealth is ‘interaction,’ covering the use of eHealth to facilitate communication between all healthcare participants. The final function of eHealth is ‘data utilization,’ referring the collection, management, and use of health and medical data sources to inform medical decision-making and intervention development [13] (Figure 1).

Thus, eHealth is not limited to mobile apps that can track a patient’s behaviour or symptoms. It encompasses communication technology that facilitates the exchange of information between primary and secondary care, or ‘big data' research that informs the development of risk assessment tools. The first two categories of eHealth technologies, those that provide opportunities to monitor and inform and those that optimize communication, are perhaps most easily linked to the daily practice of primary care providers. However, electronic data collection to enable long-term monitoring, risk detection, and research are also essential to improve primary care. For example, a large data-mining study was able to include over 50 000 primary care patients and verify if the currently used Framingham indicators for heart failure were indeed able to predict eventual heart failure cases, years before diagnosis [14].

**Returning to Jon**

Each of these three functionalities could benefit Jon. Telemonitoring, for instance, might improve control of his hypertension and diabetes. Remote consultations and home exercise programmes might facilitate his access to care [15]. Moreover, his healthcare provider’s use of the routine data could provide leads to determine the levels of service provision needed for patients like Jon [12].

### eHealth in primary care?

The speed at which the field of eHealth has expanded is unparalleled [16]. In 2017 alone, 325 000 mobile health apps were available [17], although persistence with their use was often poor [18] and quality was questionable [19,20]. Policymakers are responding to this development and urge healthcare providers to integrate the use of eHealth applications into their daily practice [7–9]. The European Commission has set ambitious goals for the implementation of eHealth in its ‘Communication on Digital Transformation of Health and Care’ [21]. Likewise, several European countries have communicated eHealth strategy plans. In Ireland, for example, the government has communicated lofty goals on eHealth implementation and has stated that by 2020 patients should ‘be able to inform themselves on health information through accredited sources of digital information’ and that care providers ‘should have the ability to monitor and interact with patients constantly despite distance and mobility of either party’ [22].

Although the use of eHealth is being stimulated, its feasibility and efficacy for primary care have only been partially demonstrated. Several studies have provided evidence that eHealth has the potential to improve primary care practice, especially concerning chronic diseases, patient self-management and patient empowerment [23]. However, no definitive conclusions can be drawn yet [24].

**Returning to Jon**

For patients like Jon, evidence on the use of eHealth to improve self-management and empowerment in hypertension and diabetes is robust [12,16]. In contrast, in the context of COPD, such evidence for telehealth is less clear [25] with a primary care-based trial showing an increase in workload and no benefit on admission rates [26].

It is, therefore, not surprising that physicians are often unsure about the quality of eHealth applications, and that they find it difficult to determine which ones to recommend to their patients [27]. For example, the Dutch national eHealth monitor 2017 provided some insight into the implementation of eHealth in primary care in the Netherlands. It revealed that while 62% of primary care practices offered possibilities for a video consultation, only 24% allowed patients to access their medication logs online, and only 11% reported that patients could access their laboratory test results [28]. These findings underline that although most GPs and patients state that they feel positive about the use of eHealth [29], they experience barriers towards its implementation and use [16].

## Conditions for developing eHealth in primary care

**Figure 2.**
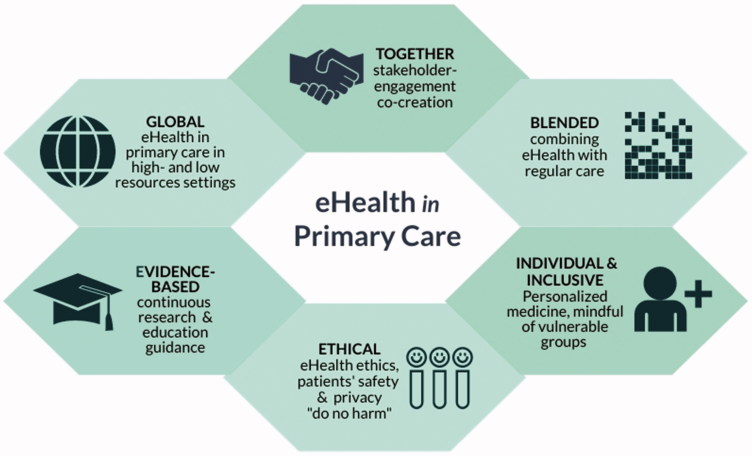
Conditions to develop and implement safe and evidence-based eHealth in primary care.

How can we go from postulated claims on the potential to empirical evidence on the (cost) effectiveness, safety and implementation of eHealth applications in primary care? We will discuss six conditions we view as vital to optimizing chances for developing and making available effective, safe and implementable eHealth applications for primary care (Figure 2).

### Together: stakeholder engagement and co-creation

Even if the idea behind an eHealth application is sound, the adoption of the application into daily practice may be challenging [30]. Uptake of an eHealth application often follows the so-called ‘hype cycle’ [31], in which adoption is propelled by an innovation trigger, reaching a peak of use, which is then transformed into a descent of disillusionment as implementation challenges become apparent. During disillusionment, a slope of enlightenment can be activated if an eHealth application is refined by stakeholder feedback. Hence, to ensure an eHealth application is successfully implemented and its use is maintained is it important to pay sufficient attention to the interaction between user demands, technology, and the intended context.

A promising strategy to achieve this is a process derived from business modelling called ‘co-creation.’ In co-creation, relevant stakeholders are invited to express their wants and needs throughout the process of eHealth development and implementation. The intervention is then changed iteratively following these wants and needs [32]. Co-creation is not only focussed on design but also on value specification. In value specification [33], developers aim to elucidate whether the intended purpose (instead of design) of the technology fits with the demands of the setting. For instance, when developing a teledermatology application, healthcare professionals and patients not only provided comments on the application itself, but also expressed the need for a practitioner guide on how to use the application. Without such a guideline, healthcare professionals felt that the application would be of little value to their hospital [34]. Another promising tool to guide both the content and design of eHealth applications is scenario-based prototype co-creation, in which all those involved are nudged to express and visualize their work routines and the problems they encounter [35,36].

In general, co-creation techniques have been demonstrated to improve the (continued) implementation of eHealth applications and their levels of acceptability and feasibility [37]. A study on the co-creation process of a web-based platform for DM2 patients and their care providers revealed that views and preferences regarding the platform differed significantly between patients and providers [38]. For instance, providers were set against the wish of patients to restrict access to certain information on the platform and expressed contrasting ideas on the use of medication alerts. Considering these possibly opposing views by ensuring substantial and iterative engagement of intended user groups might, therefore, be a vital prerequisite for eHealth success in primary care.

### Blended: combining eHealth with regular care

Most eHealth applications are used by individuals, without the assistance or guidance of a care provider. However, ‘blended care,’ in which face-to-face care is combined with eHealth applications, is becoming increasingly popular. Blended care has the potential to improve the quality and efficiency of care, while maintaining—or even improving—patient and provider satisfaction with care. For example, the implementation of a blended care approach for patients suffering from medically unexplained symptoms led to reduced unnecessary medical consumption and improved work participation [39].

**Returning to Jon**

Imagine Jon, who is trying to quit smoking with the support of the smoking cessation app *My Quit Coach*. Research has shown that blending online (e.g. an app to stimulate patients’ self-management) and offline (e.g. counselling provided by a practice nurse) care elements can improve adherence to treatment and patients’ health status [40,41]. As an example, Jon’s use of the app *My Quit Coach* might benefit from the encouragement of his practice nurse, who is prompted by the electronic health record to ask about his quit attempt during a video consultation [42].

Research on the use of ‘blended care’ is still relatively small, but the first results are promising [41]. For instance, a recent study that evaluated the combination of a web-based risk assessment tool and face-to-face coaching sessions for obese patients; the blended approach showed promising effects on weight loss among at-risk populations [43]. Adding to this, a recent study investigating the impact of a blended care approach launched by the Dutch College of General Practitioners found that the use of a non-commercial, evidence-based website combined with advice provided during regular, face-to-face care related to a 12% decrease in primary care consultations after two years [44]. More research has to be performed to demonstrate how, and to what degree, blended care can be beneficial to the primary care setting. The limited evidence available suggests, however, that practising blended care may save a significant amount of (consultation) time, which can be redirected towards high-quality face-to-face care for those patients that need it the most.

### Individualized and inclusive

Personalized eHealth appears to be more effective than those applications that apply a one-size-fits-all approach [45]. This finding underlines the need for a more holistic and tailored approach to eHealth, in which individual needs are taken into account and applications are adapted accordingly [46]. This personalized approach fits well with the person-centred care as practised by primary care professionals for decades. In personalized (eHealth) medicine, patient characteristics ranging from genes to environmental factors inform the formulation of a tailored care plan or strategy for the individual patient. To gain insight into patient characteristics, the use of machine learning represents a growing area of interest [47]. Machine learning can be used to inform diagnostic processes but also to predict treatment responses and to individualize treatment [48,49]. For instance, machine-learning algorithms can be used to predict glycaemic responses to meals in patients with DM2 and help establish personalized diet plans [50].

**Returning to Jon**

Standardized algorithms for detecting exacerbations of COPD are little better than chance [51], but machine learning may enable the development of an algorithm that can predict when his COPD will exacerbate and enable timely action [52].

These applications of machine learning in healthcare underline the importance of viewing eHealth as a triad in which ‘data utilization’ (the collection, management, and use of health data sources) is of equal importance as those eHealth applications that inform, monitor, track, and interact. To individualized eHealth applications, the focus should also be on inclusiveness. eHealth has the potential to both increase and decrease health inequalities [53]. Most eHealth applications require users to have a fair degree of (health) literacy, and a moderate understanding of and proficiency with digital technology. When eHealth gives rise to health inequalities, this is often linked to a worsening of existing differences, and not a newly introduced form of inequality [54]. We argue that eHealth developers and implementers should be aware of this potential risk to exacerbate inequity, and would urge them to implement strategies to optimize inclusiveness of groups at risk. Embedding iterative co-creation processes with vulnerable groups during eHealth development and implementation, and tailoring applications by the users’ level of (digital) literacy might help to reduce health inequalities [51].

***Returning to Jon***

Jon suffers from diabetes-related cataracts. For him, being shown how to increase the font size on a tablet might be a crucial facility in using such eHealth technology.

### Global: eHealth in primary care in high- and low-resource settings

eHealth technology is not only relevant to high-resource settings; primary care in lower resource settings could also benefit from the use of eHealth [55]. In these settings, the patient-to-doctor ratio is often high. For instance, in Kenya, only one doctor is available per 5000 patients [56]. In addition, the average distance to the nearest primary healthcare facility in these settings is mostly long. However, the majority of people do own a mobile phone and can receive text messages or use apps. Hence, there is ample opportunity to improve healthcare using eHealth. For instance, local healthcare workers might be able to provide routine medical care to patients supported by eHealth applications that give medical decision support or a long distance teleconnection with doctors in secondary care facilities.

Non-communicable, chronic diseases are on the rise in low resource settings [57]. Especially for those diseases, eHealth could be an efficient and valuable instrument to increase the reach and impact of self-management interventions [58–60]. For example, a recent RCT examining the effect of a mobile health self-management intervention in DM2 patients in Bangladesh revealed a significant increase in glycaemic control in the intervention group after six months [61].

eHealth might also be utilized to enhance healthcare accessibility. In rural China, for example, the long distance to healthcare facilities is a significant problem. So-called ‘internet hospitals’ allow patients to receive high-quality care from a top-tier hospital from either their own home or a local clinic, through a video or telephone connection [62].

Although the potential of eHealth to affect primary care in low resource settings is high, eHealth development and research is mostly focussed on, and performed, in high resource settings. Because of the major impact eHealth could have on healthcare systems in low resource settings, we argue that (research on) the upscaling of eHealth applications to those settings should be considered and prioritized.

### Evidence-based: continuous research and educational guidance

Hundreds of thousands of eHealth applications are available online and in app stores. However, for most of these applications, we do not know whether they are safe or (cost)-effective [63]. And if there is knowledge available, it mostly shows that the quality and effectivity of eHealth applications is still suboptimal or under-researched. For example, a recent content analysis of apps for hypertension concluded that most were of poor quality [64].

**Returning to Jon**

So, without guidance, Jon’s primary care clinician may find it difficult to advise a patient such as Jon on a suitable app to help monitor his blood pressure.

An important reason for this evidence gap is the lack of available research strategies that can keep up with the pace in which eHealth applications are developed and amended while maintaining scientific rigour [65]. When using traditional research designs, such as the randomized controlled trial (RCT), it takes four to five years on average to finish the trial and publish the outcome. This time window issue makes most traditional research designs less suitable for the evaluation of eHealth applications; by the time results are available, the application studied would be outdated. To illustrate; in four years, we went from using a cell phone equipped with short message service (SMS) in 2005 to using a 3G smartphone running WhatsApp in 2009.

**Figure 3.**
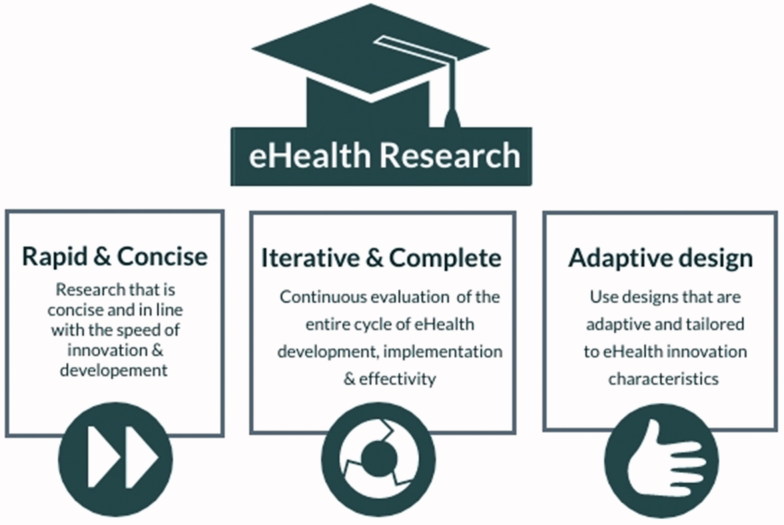
Characteristics of eHealth research.

The pace in which eHealth applications are developed and implemented calls for innovative research designs, which are both rapid and concise. Moreover, it calls for the use of research designs that take into account the dissemination and implementation of the application during the research process and can be adapted iteratively when needed. Glasgow et al., [66] introduced such a design, called the ‘rapid and relevant research paradigm,’ which has the potential to accelerate the eHealth research process without decreasing research quality. In this design, research starts with a one-to-three-month period in which a rapid literature and best practices assessment is performed, followed by the process of fast prototyping and refinement of the intended application. After that, an exploratory evaluation (also known as ‘evaluability assessment’ [67]) is conducted to determine the likelihood of finding a positive effect of the application in practice. If this evaluation has a positive outcome, it should be followed by several concise ‘experiments’ following different designs such as n-of-1 [68], A-B quasi-experimental [69], multiphase optimization strategy (MOST) [70], interrupted time series (ITS) [71] or the sequential multiple assignment randomized trial (SMART) [72]. This phase should take between two and six months in total. If the application is determined to be potentially feasible and effective, another six months will be spent on performing a larger trial, preferably based on a stepped-wedged or comparative effectiveness research (CER) design, to provide evidence on efficacy [65]. Research should not stop there, however. Widespread implementation of the eHealth application should be guided by continuous cycles of assessment and improvement based on the input of stakeholders and advancement in technologies. Figure 3 summarizes the characteristics of eHealth research.

To facilitate the uptake of eHealth in practice further, the focus should be on education and the formalization of the learning process of (future) primary care providers on the use of eHealth [73]. As such, integrating eHealth learning programmes into the medical curriculum is suggested as a key factor in the successful implementation of eHealth in primary care [74]. For example, a recent study performed in Zurich demonstrated that after the addition of a module on ‘clinical telemedicine and eHealth’ to the medical curriculum, 93% of students indicated they would use eHealth in their future practice as a physician [75]. Finally, another innovative way to increase some patients’ engagement with and uptake of eHealth application is incorporating features such as gamification and telepresence [76]. For example, a ‘serious digital game’ provided to 47 women who were contemplating diet changes, led to a significant increase in nutritional knowledge and decrease in body mass index (BMI) after three months [77].

### Ethical: being attentive of ethical considerations, privacy and patient safety

The first rule of medicine is to ‘do no harm’ [78]. Hence, all healthcare providers need to ensure that the innovations or treatments they implement do not cause any adverse or unintended effects among the patient population. As eHealth involves the use of technology, ‘do no harm’ is not only to be embedded in medical actions but also acknowledged when considering the safety and reliability of technological innovations. For instance, the protection of users’ (online) privacy and data—how can we ensure that personal data collected via eHealth applications is anonymized correctly and stored safely so that the applications or its users will not become victims of cyber-crime? And how can we adequately manage the rising commercial interest in health data from influential companies, such as Google or Apple?

Several strategies have been proposed to maximize data protection and user privacy [79]. The Horizon2020 KONFIDO project, for instance, provides several tools to optimize the safety and resilience of eHealth data storage and exchange systems [80]. Apart from technological safety, it is also essential to be mindful of the medical consequences of eHealth use at large. Although studies have demonstrated that eHealth can have beneficial effects, some adverse effects have also been reported [81]. In particular, adverse effects of eHealth use have been related to socioeconomic status and (e)health illiteracy of its users. Younger, healthier and more highly educated individuals most frequently use eHealth. Hence, following this statement, there is a real risk that eHealth improves the health status of the so-called ‘worried-well’, and not that of vulnerable, high-risk groups. In that sense, eHealth has the potential to increase health inequality [53]. Offering technology that contains meaningful messages tailored towards low-literate populations can help to improve general accessibility, and screening tools on health (il)literacy might be able to enhance identification and handling of low-literacy from the outset [82]. Taking these matters into account, we advise (1) putting ‘do no harm’ high on the eHealth (research) agenda, for instance, by including a skilled ethicist in the team who is familiar with eHealth, and (2) to implement co-creation processes with vulnerable groups that can provide vital information on how to make eHealth applications suitable and feasible for all those in need.

## The future of eHealth in primary care

Indications that eHealth has the potential to improve primary care practice are present. eHealth may be able to help address the steady rise of patients presenting with multimorbidity in primary care. Moreover, it may help support the transition towards personalized medicine, self-management and shared decision-making in primary care. As illustrated in the case of ‘Jon,’ eHealth could potentially make a real difference to his health and wellbeing. Several steps forward, however, need to be made to ensure safe and cost-effective eHealth applications become available to patients like Jon and their caregivers, and all others involved in primary care. These include active stakeholder engagement and co-creation, the use of personalized and blended care approaches that prioritize inclusivity and health equality and using adaptive (action research) designs to rapidly generate robust evidence either for or against the use of specific eHealth innovations. In addition, developers and clinicians should be mindful both of ethical and of safety considerations. Furthermore, there should be a focus on the application of eHealth for primary care in low resource settings, where the potential for improvement via eHealth may be even greater than in high resource settings [55,83].

eHealth living-labs facilitate a patient-centred, evidence-based milieu for innovation development and implementation. They provide an opportunity to bring together relevant eHealth stakeholders and initiate processes of co-creation, human-centred design and action research [84]. By stimulating an open-source development of eHealth on a national level, the establishment of high quality, evidence-based eHealth solutions that solve health(care) problems experienced by patients and healthcare providers is facilitated. Moreover, as these labs enable interdisciplinary collaboration between patients, practitioners, public and private organizations, policymakers, educational professionals and scientists; they can help to establish a local and national mandate for the uptake and formal ratification of eHealth in primary care.

## Conclusion

eHealth holds great potential for primary care. However, several barriers, such as innovation complexity and lack of evidence on eHealth effectivity, need to be addressed before eHealth can be implemented in primary care routines. Moreover, awareness of the benefits and downsides of eHealth needs to improve to enable primary care providers to make informed decisions on the usability and application of eHealth in their practices. eHealth can support—and may even transform—the primary care landscape, but a collaborative effort between science and practice is needed to experiment with eHealth design and implementation, learn from our experiences, and adequately document the results and subsequent recommendations formulated.
